# Supplementary material for: HEXIM1 inter-monomer autoinhibition governs 7SK RNA binding specificity and P-TEFb inactivation
Source: Nat Commun. 2026 Jan 15;17:1570. doi: 10.1038/s41467-026-68285-8 (PMC12901311; doi:10.1038/s41467-026-68285-8)
Supplement: Supplementary file 2 — Reporting Summary [file 41467_2026_68285_MOESM2_ESM.pdf]

nature portfolio

Corresponding author(s): Yuan Yang, Juli Feigon

Last updated by author(s): Dec 17, 2025

Reporting Summary

Nature Portfolio wishes to improve the reproducibility of the work that we publish. This form provides structure for consistency and transparency in reporting. For further information on Nature Portfolio policies, see our Editorial Policies and the Editorial Policy Checklist.

Statistics

For all statistical analyses, confirm that the following items are present in the figure legend, table legend, main text, or Methods section.

n/a	Confirmed	
☐	☒	The exact sample size (*n*) for each experimental group/condition, given as a discrete number and unit of measurement
☐	☒	A statement on whether measurements were taken from distinct samples or whether the same sample was measured repeatedly
☒	☐	The statistical test(s) used AND whether they are one- or two-sided<br>*Only common tests should be described solely by name; describe more complex techniques in the Methods section.*
☒	☐	A description of all covariates tested
☐	☒	A description of any assumptions or corrections, such as tests of normality and adjustment for multiple comparisons
☐	☒	A full description of the statistical parameters including central tendency (e.g. means) or other basic estimates (e.g. regression coefficient) AND variation (e.g. standard deviation) or associated estimates of uncertainty (e.g. confidence intervals)
☒	☐	For null hypothesis testing, the test statistic (e.g. *F*, *t*, *r*) with confidence intervals, effect sizes, degrees of freedom and *P* value noted<br>*Give P values as exact values whenever suitable.*
☒	☐	For Bayesian analysis, information on the choice of priors and Markov chain Monte Carlo settings
☒	☐	For hierarchical and complex designs, identification of the appropriate level for tests and full reporting of outcomes
☒	☐	Estimates of effect sizes (e.g. Cohen's *d*, Pearson's *r*), indicating how they were calculated

Our web collection on statistics for biologists contains articles on many of the points above.

Software and code

Policy information about availability of computer code

Data collection	All relevant data collection softwares are also listed in the relevant Methods section.<br>Mass Photometry: Refeyn AcquireMP<br>NMR data collection: TopSpin versions 1, 2 and 3<br>ITC data collection: MicroCal ITC200
Data analysis	All relevant data analysis softwares are also listed in the relevant Methods section.<br>Mass Photometry: Refeyn DiscoverMP<br>Gel image analysis: ImageJ 1.54g<br>EMSA fitting and NMR data display: Igor-Pro 9<br>NMR data processing: NMRpipe versions 8.9 Rev 2017.047.15.28 64-bit and 10.9 Rev 2021.258.11.26 64-bit<br>NMR data analysis: NMRFAM-Sparky 1.414<br>ITC data analysis: ORIGIN 7 (Micro-Cal)<br>Sequence alignment analysis: Jalview version 2.11.5.1

For manuscripts utilizing custom algorithms or software that are central to the research but not yet described in published literature, software must be made available to editors and reviewers. We strongly encourage code deposition in a community repository (e.g. GitHub). See the Nature Portfolio guidelines for submitting code & software for further information.

Data

Policy information about availability of data

All manuscripts must include a data availability statement. This statement should provide the following information, where applicable:

- Accession codes, unique identifiers, or web links for publicly available datasets
- A description of any restrictions on data availability
- For clinical datasets or third party data, please ensure that the statement adheres to our policy

Accession Numbers
Backbone chemical shift assignments of BR-L-AR protein, and backbone/sidechain chemical shift assignments of SL1-dIU RNA-bound BR-L-AR protein have been deposited in the Biological Magnetic Resonance Data Bank (BMRB), under accession IDs 53441 [https://dx.doi.org/10.13018/BMR53441] and 53442 [https://dx.doi.org/10.13018/BMR53442], respectively. Backbone and sidechain chemical shift assignments of additional monomeric protein constructs BR-L and BR have been deposited in BMRB under accession IDs 53446 [https://dx.doi.org/10.13018/BMR53446]
and 53447 [https://dx.doi.org/10.13018/BMR53447], respectively. Proton chemical shift assignments of RNA constructs, SL1-dI (with additional carbon chemical shifts), SL1-dIU, SL1-dII, SL1-d, SL1-dIIm, SL1-p, SL1-mp, SL1-pII and SL1altUUCG, have been deposited in BMRB under accession IDs 53443/53444 [https://dx.doi.org/10.13018/BMR53443] [https://dx.doi.org/10.13018/BMR53444], 53445 [https://dx.doi.org/10.13018/BMR53445], 53448 [https://dx.doi.org/10.13018/BMR53448], 53449 [https://dx.doi.org/10.13018/BMR53449], 53450 [https://dx.doi.org/10.13018/BMR53450], 53451 [https://dx.doi.org/10.13018/BMR53451], 53452 [https://dx.doi.org/10.13018/BMR53452], 53453 [https://dx.doi.org/10.13018/BMR53453], 53454 [https://dx.doi.org/10.13018/BMR53454], respectively.
Data Availability
Raw data analyses are included as a combined supplementary spreadsheet for protein CSPs, protein PRE intensity ratios, and RNA CSPs, respectively.

Research involving human participants, their data, or biological material

Policy information about studies with human participants or human data. See also policy information about sex, gender (identity/presentation), and sexual orientation and race, ethnicity and racism.

Reporting on sex and gender	N/A
Reporting on race, ethnicity, or other socially relevant groupings	N/A
Population characteristics	N/A
Recruitment	N/A
Ethics oversight	N/A

Note that full information on the approval of the study protocol must also be provided in the manuscript.

Field-specific reporting

Please select the one below that is the best fit for your research. If you are not sure, read the appropriate sections before making your selection.

- [x] Life sciences
- [ ] Behavioural & social sciences
- [ ] Ecological, evolutionary & environmental sciences

For a reference copy of the document with all sections, see nature.com/documents/nr-reporting-summary-flat.pdf

Life sciences study design

All studies must disclose on these points even when the disclosure is negative.

Sample size	Three to four independent experiments with good agreement within each group are performed for ITC binding experiments.
Data exclusions	No data were excluded from the analyses.
Replication	All replicates were performed in independent measurements. Additional control ITC experiments with wild-type protein and RNA from two or more batches of protein purified show good reproducibility.
Randomization	The order of protein substitutes used is randomized and shows no correlation between order of usage and resulting binding affinities.
Blinding	Not applicable to the study as experimental conditions are well controlled and all biochemical reagents are clearly labeled.

Reporting for specific materials, systems and methods

We require information from authors about some types of materials, experimental systems and methods used in many studies. Here, indicate whether each material, system or method listed is relevant to your study. If you are not sure if a list item applies to your research, read the appropriate section before selecting a response.

Materials & experimental systems

n/a	Involved in the study	
☒	☐	Antibodies
☒	☐	Eukaryotic cell lines
☒	☐	Palaeontology and archaeology
☒	☐	Animals and other organisms
☒	☐	Clinical data
☒	☐	Dual use research of concern
☒	☐	Plants

Methods

n/a	Involved in the study	
☒	☐	ChIP-seq
☒	☐	Flow cytometry
☒	☐	MRI-based neuroimaging

Plants

Seed stocks	N/A
Novel plant genotypes	N/A
Authentication	N/A
